# Wound infection following implant removal of foot, ankle, lower leg or patella; a protocol for a multicenter randomized controlled trial investigating the (cost-)effectiveness of 2 g of prophylactic cefazolin compared to placebo (WIFI-2 trial)

**DOI:** 10.1186/s12893-020-01024-y

**Published:** 2021-02-01

**Authors:** Fay R K Sanders, Diederick Penning, Manouk Backes, Siem A Dingemans, Susan van Dieren, Anne M Eskes, J Carel Goslings, Peter Kloen, Ron A A Mathôt, Niels W L Schep, Ingrid J B Spijkerman, Tim Schepers

**Affiliations:** 1 Trauma Surgery, Amsterdam UMC, Loc. AMC, G4-137, Meibergdreef 9, 1105 AZ Amsterdam, The Netherlands; 2 Trauma Surgery, OLVG, Loc. West, Jan Tooropstraat 164, 1061 AE Amsterdam, The Netherlands; 3 Orthopedic Surgery, Amsterdam UMC, Loc. AMC, Meibergdreef 9, 1105 AZ Amsterdam, The Netherlands; 4 Hospital Pharmacy, Amsterdam UMC, Loc. AMC, Meibergdreef 9, 1105 AZ Amsterdam, The Netherlands; 5 Trauma Surgery, Maasstad Ziekenhuis, Maasstadweg 21, 3079 DZ Rotterdam, The Netherlands; 6 Medical Microbiology, Amsterdam UMC, Loc. AMC, Meibergdreef 9, 1105 AZ Amsterdam, The Netherlands

**Keywords:** Trauma surgery, Orthopedic surgery, Implant removal, Antibiotic prophylaxis, Cefazolin, Surgical site infections, Lower extremity, Foot, Ankle

## Abstract

**Background:**

Elective implant removal (IR) after fracture fixation is one of the most common procedures within (orthopedic) trauma surgery. The rate of surgical site infections (SSIs) in this procedure is quite high, especially below the level of the knee. Antibiotic prophylaxis is not routinely prescribed, even though it has proved to lower SSI rates in other (orthopedic) trauma surgical procedures. The primary objective is to study the effectiveness of a single intravenous dose of 2 g of cefazolin on SSIs after IR following fixation of foot, ankle and/or lower leg fractures.

**Methods:**

This is a multicenter, double-blind placebo controlled trial with a superiority design, including adult patients undergoing elective implant removal after fixation of a fracture of foot, ankle, lower leg or patella. Exclusion criteria are: an active infection, current antibiotic treatment, or a medical condition contraindicating prophylaxis with cefazolin including allergy. Patients are randomized to receive a single preoperative intravenous dose of either 2 g of cefazolin or a placebo (NaCl). The primary analysis will be an intention-to-treat comparison of the proportion of patients with a SSI at 90 days after IR in both groups.

**Discussion:**

If 2 g of prophylactic cefazolin proves to be both effective and cost-effective in preventing SSI, this would have implications for current guidelines. Combined with the high infection rate of IR which previous studies have shown, it would be sufficiently substantiated for guidelines to suggest protocolled use of prophylactic antibiotics in IR of foot, ankle, lower leg or patella.

*Trial registration* Nederlands Trial Register (NTR): NL8284, registered on 9th of January 2020, https://www.trialregister.nl/trial/8284

## Background

In the Netherlands, each year, about 18,000 surgical implants are removed after fracture healing [1]. Most fracture implant removals (IR) are performed in the lower extremity (85.7% of all IR surgeries) and the removal rate is the highest in foot and ankle [2, 3]. A surgical site infection (SSI) is one of the most common complications of surgical interventions of the lower extremity, especially when implants are involved. The infection rate ranges from 1.3 to 10% in hip and knee procedures [4, 5] to 4.5–24.6% in foot and ankle surgery [6–8]. SSIs are not only responsible for prolonged hospital stay and a significant increase in healthcare costs, the functional outcome of patients who suffered from an infection is also relevantly decreased [9]. Even though IR is regarded a “clean procedure” according to the Centers for Disease Control and Prevention (CDC) classification of surgical wounds [10], and it usually is a relatively short procedure (< 1 h), the infection rate of 8–20% [11, 12] is at least as high as in procedures where orthopedic implants are placed.

As IR is classified as a “clean procedure” in current guidelines, prophylactic antibiotics are not routinely administered [13]. Previously, a randomized controlled trial (WIFI trial) investigated the effect of 1 g of cefazolin on SSIs in IR below the level of the knee and found that overall infection rates did not significantly decrease (14.9% vs. 13.2%) [14]. However, there was a trend towards a lower rate of deep SSIs with resp. 2.9% in placebo and 0.4% in the cefazolin group.

Recently, guidelines on general surgical prophylaxis have been revised, now suggesting 2 g instead of 1 g of cefazolin for implant surgery taking longer than 1 h and 3 g for obese individuals (body mass index (BMI) > 40)[13, 15]. The decision to increase the dosage from 1 to 2 g was mostly based on pharmacological studies [16, 17]. Although the difference in tissue concentrations between the two dosages has also been investigated in orthopedic trauma surgery, the clinical effect of a higher dosage remains unclear [18]. To the best of our knowledge, only one recent retrospective cohort study compared 2 g with 1 g of prophylactic cefazolin on the incidence of SSIs in foot and ankle surgery. This study could not demonstrate a statistically significant difference with 4.8% vs. 6.5% SSIs, but did conclude that the difference might be clinically relevant. In gynecology, a large retrospective study reported that 2 g was associated with a significantly lower risk of SSI compared to 1 g of cefazolin (OR 0.967, 95% CI 0.94–0.99) [19].

Our hypothesis is that prophylactic cefazolin in a dose of 1 g does not sufficiently penetrate the more distal parts of the lower extremity [18]. This hypothesis is supported by studies measuring cefazolin concentrations in both hip and knee bone, which found significantly lower values in the knee than in the hip after prophylactically administered cefazolin [20, 21]. Deacon et al. reported even lower concentrations in the foot [22]. A single dose of 2 g of cefazolin would increase not only the duration of adequate coverage but also the peak concentration.

Given the high rate of SSIs in IR and evidence that higher dosages are required below the level of the knee, we feel that there are sufficient arguments to evaluate whether 2 g of cefazolin is effective as prophylaxis. Therefore, the primary objective of this randomized controlled superiority trial is to study the effectiveness of a single intravenous dose of 2 g of cefazolin on SSIs prior to IR following fixation of foot, ankle, lower leg or patella fractures.

Secondary objectives are to study the cost-effectiveness of 2 g of cefazolin preventing SSIs after IR (only when a statistically significant effect is found); to elucidate the underlying mechanism of antibiotic prophylaxis by measuring target-site concentrations of cefazolin; to identify possible underlying infections (before IR); and to identify independent predictors of SSI.

## Methods

This is a multicenter, randomized double-blind placebo controlled intervention trial with a superiority design, comparing 2 g of cefazolin as antibiotic prophylaxis to placebo with a 1:1 allocation ratio.

### Participants

The trial will run in approximately 20 hospitals in the Netherlands, both academic and non-academic centers. A list of participating sites can be found on the trial website: https://www.amc.nl/web/research-75/trials-collaborations/wifi-2.htm.

All consecutive patients (age 18–75), scheduled for elective IR in foot, ankle, lower leg or patella are eligible for inclusion.

Exclusion criteria are:

- Removal and re-implantation of osteosynthesis material in the same session
- Active wound infection or antibiotic treatment (for any reason) at time of IR
- A medical history of serious peripheral vascular disease, severe renal insufficiency
- Allergy for cephalosporin, or severe allergy for penicillin/other beta-lactam antibiotic
- Treatment with probenecid or immunosuppressants
- Pregnancy
- Insufficient comprehension of Dutch/English language

### Randomization

After signing informed consent forms, patients will be randomly assigned to the intervention or control group (1:1 allocation, random block sizes of 2, 4 or 6), using a computerized randomization module stratified by academic/non-academic center to ensure allocation concealment. Randomization will be performed preoperatively by the coordinating investigator using a dedicated, password protected, SSL–encrypted website (Castor) and the responsible anesthesiologist will be notified of the result, while being unaware of allocation sequence. If the electronic randomization module fails for any reason, randomization will be performed by tossing a coin (head signifying cefazolin and tails placebo).

### Blinding

The patient, operating (orthopedic) surgeon and outcome assessors will all be blinded for the result of randomization. Unblinding will not be performed until the end of the trial. If the attending physician does decide that unblinding is necessary, (s)he will make every effort to contact the coordinating investigator before unblinding to discuss options. Statistical analysis will be performed by an independent researcher, blinded for the randomization result. The randomization code will be unblinded after complete analysis of the study results.

### Interventions

If patients are allocated to the intervention group, they will receive a single dose of either 2000 mg or 3000 mg of cefazolin solved in 10 cc of Sodium Chloride (NaCl) 0.9% through a peripheral intravenous (iv) catheter. The actual dose is dependent on the patient’s BMI; patients with a BMI over 40 will receive 3000 mg instead of 2000 mg of cefazolin. The drug will be prepared by the anesthesiologist/assistant and administered in the theatre/holding-area within 60 min prior to surgery. When allocated to the placebo/control group, the patient receives a single dose of 10 cc of Sodium Chloride (NaCl) 0.9% in the same manner. Both will be administered in absence of the surgeon to avoid unblinding. The anesthesiologist/assistant then fills out a form containing: randomization number, date, placebo/ cefazolin dose (2 g/3 g), LOT number, expiration date and the initials of the anesthesiologist. This form will be sealed in a closed envelope and sent to the coordinating investigator. The envelope will remain sealed until the end of the study period and is opened at the time of analysis to check whether the patient received the allocated drug/correct dosage.

The exact timing of antibiotic administration and surgical technique and characteristics are not predefined, since this is a pragmatic trial, designed to resemble daily practice as much as possible. However, these characteristics are all collected to use in the multivariable analysis predicting the risk of SSI.

### Outcomes

The primary outcome parameter is SSI within 90 days, as defined by the criteria used in the latest CDC guideline for the prevention of SSI (Table 1). The criteria for a superficial infection will however be modified to the extent where diagnosis by surgeon/attending will have to be confirmed by an independent expert. Therefore, a picture and description of the wound will be required when the physician suspects an infection.

**Table 1:** CDC surgical site infection criteria [25]

*Superficial incisional SSI*: Date of event for infection occurs within 30 days after the operative procedure (where day 1 = the procedure date) AND involves only skin and subcutaneous tissue of the incision AND patient has at least one of the following:	
a	Purulent drainage from the superficial incision
b	Organisms identified from an aseptically-obtained specimen from the superficial incision or subcutaneous tissue by a culture or non-culture based microbiologic testing method
c	Superficial incision that is deliberately opened by a surgeon, attending physician or other designee AND culture or non-culture based testing is not performed AND patient has at least one of the following signs or symptoms: pain or tenderness; localized swelling; erythema; or heat
d	Diagnosis of a superficial incisional SSI by the surgeon or attending physician or other designee
*Deep incisional SSI:* Must meet the following criteria: The date of event for infection occurs within 90 days after the operative procedure (where day 1 = the procedure date) AND involves deep soft tissues of the incision (for example, fascial and muscle layers) AND patient has at least one of the following:	
a	Purulent drainage from the deep incision
b	A deep incision that spontaneously dehisces, or is deliberately opened or aspirated by a surgeon, attending physician or other designee AND organism is identified by a culture or nonculture based microbiologic testing method; or culture or non-culture based microbiologic testing method is not performed AND patient has at least one of the following signs or symptoms: fever (> 38 °C); localized pain or tenderness. A culture or non-culture based test that has a negative finding does not meet this criterion
c	An abscess or other evidence of infection involving the deep incision that is detected on gross
	Anatomical or histopathologic exam, or imaging test

Secondary outcomes are:

- Other infectious outcomes possibly related to the surgical procedure. These include wound dehiscence (without qualifying as a superficial SSI) and S. aureus bacteremia (which does not qualify as a SSI after extensive assessment for a focus of infection). This outcome does not include hospital acquired infections such as pneumonia or urinary tract infections.
- Cost-effectiveness of intervention: measured with health care resource utilization and costs (iMCQ, iPCQ); at baseline, 6 weeks, 3 months and 6 months after surgery. Cost effectiveness will be measured as cost per patient free of SSI and the cost utility analysis will be described as cost per quality adjusted life years (QALY’s). QALYs will be measured by the 5-level EuroQuality of Life-5D (EQ-5D-5L); at baseline, 2 weeks, 6 weeks, 3 months and 6 months after surgery.
- Target-site antibiotic concentrations: the time that target site concentrations of prophylactic cefazolin (μg/L) stay above the minimal inhibitory concentration needed to adequately prevent SSIs (T > MIC) will be measured during surgery from at least two samples at varying time points in blood and soft tissue from incision site. In addition, the concentration of cefazolin will be measured in serum, to construct a multi-compartment pharmacokinetic model (only selected patients from main research center).
- Fracture related infections: diagnosed by analyzing the presence of pathogens on removed implants, determined by culture of removed material (only Amsterdam UMC); directly following surgery. A minimum of two positive samples are required to score low-graded pathogens and one positive sample for other pathogens.
- Independent predictors of SSI, measured by a multivariate regression analysis/subgroup analysis of patient and treatment characteristics (e.g. weight, smoking).

### Data collection

#### Invasive procedures

To administer the antibiotics or placebo intravenously, a peripheral intravenous (IV) catheter is required. However, this is standard procedure during surgery because the IV-catheter is already used for either sedatives, muscle relaxants and/or pain medication.

For the additional measurements, in a selection of patients from the Amsterdam UMC, location AMC only, samples are acquired during the procedure to measure antibiotic concentrations. Samples will be taken from:

- Serum (from IV catheter, 3–4 samples at different time intervals)
- Soft tissue (from target-site, 2 samples at different time intervals)
- Blood (from target-site, 2 samples at different time intervals)

The serum samples are drawn from a second IV catheter, placed under general anesthesia. Samples from the target-site (operated foot/ankle) will be taken after incision. They are comprised of “fresh” blood that spontaneously surfaces from the bone during surgery and soft-tissue nearest to the incision. Serum will be separated from blood cells within one hour after withdrawal and stored at − 80 °C until analysis. By measuring concentrations in the samples at varying time intervals through a validated laboratory analysis, we will be able to construct individual time-concentration curves of cefazolin. Based on a pharmacokinetic multi-compartment model, fT > MIC at the target-site (secondary outcome measure) can be extracted.

#### Questionnaires

**Fig. 1:**
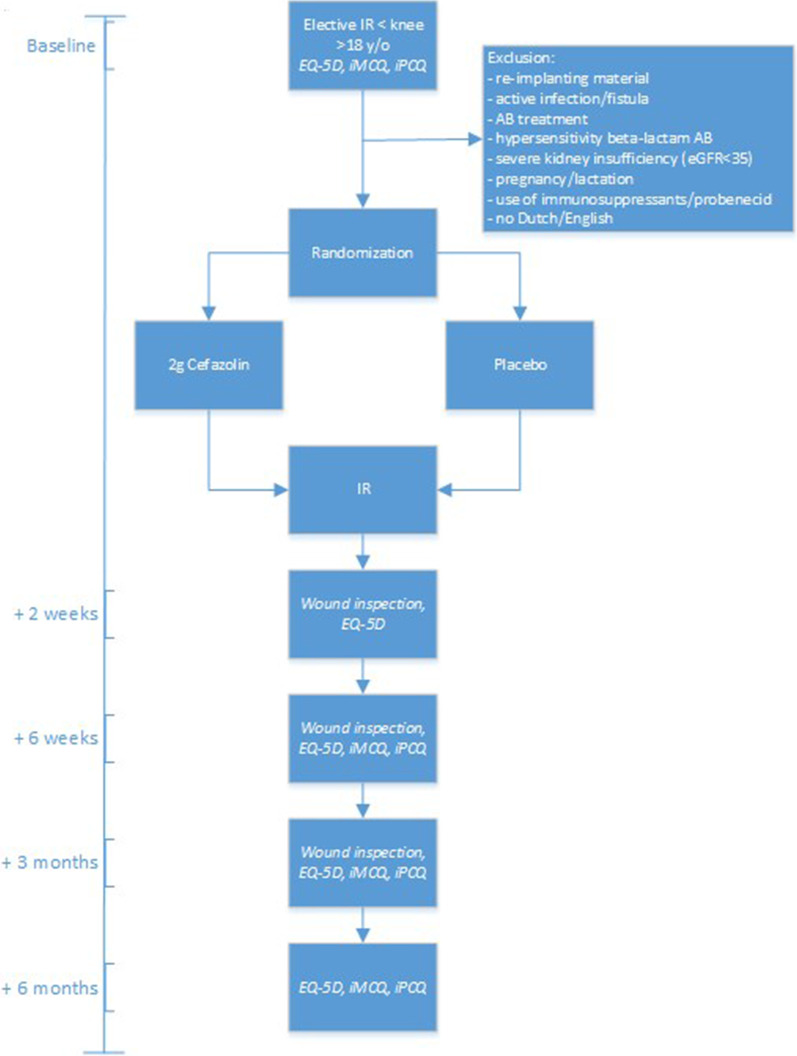
Timeline

As shown in Fig. 1, after informing the patient about the study and obtaining informed consent in the outpatient clinic, patients will be asked for a pre-operative, baseline assessment 14–1 day(s) before IR by means of 3 questionnaires they will receive by (e-)mail. Additionally, patients will fill out questionnaires at 2 weeks, 6 weeks, 3 months and 6 months after IR. These questionnaires consist of the 5-level EuroQuality of Life-5D (EQ-5D-5L) and the Dutch iMTA Medical Consumption Questionnaire (iMCQ) and iMTA Productivity Cost Questionnaire (iPCQ). In addition to these validated questionnaires, patients will be asked if they have any complaints that could suggest wound complications (combined with questionnaires at 2 weeks, 6 weeks and 3 months) or side-effects of the investigational product (only at 2 weeks).

#### Additional data collection

Patient, fracture and surgical characteristics will be collected and documented in the online, password protected, SSL-encrypted database (Castor EDC [23]). Patient characteristics comprise age, gender, weight, BMI, American Society of Anesthesiologists (ASA)-classification, substance abuse (smoking, alcohol, drugs) and medical history (including diabetes mellitus). Fracture characteristic comprise the type of fracture and the conditions of the soft tissues (open/closed) prior to fixation. Surgical characteristics comprise timing of administration of cefazolin/placebo, duration of surgery, use of a tourniquet, placement of implants, type of implants and wound closure technique. Moreover, patients and the operating surgeon will be asked for the reason for IR.

### Sample size

A total SSI rate of 14.9% is assumed in the control group, as was found in the WIFI-trial [14]. In the intervention group we aim for a 50% reduction to 7.45%, based on the Dutch Trauma Trial [24], who showed a reduction of SSIs of over 50% in a large cohort of 2195 patients with fractures of the extremity (control group: 8.3%, antibiotic prophylaxis group: 3.6%). In total, 554 patients are required to have an 80% chance of detecting a reduction from 14.9% in the control group to 7.45% in the experimental group with a chi-square test with a two-sided alpha level of 0.05. However, the WIFI-1 trial had insufficient power to demonstrate a significant difference in deep SSIs [14]. Because deep SSIs often have the most serious consequences, an intervention that reduces these infections (even if they are less common) may still be cost-effective. Therefore, we chose to expand the number of included patients in order to have sufficient power to demonstrate a difference in deep SSIs as well. Combining the number of deep SSIs of the WIFI-1 trial and an RCT by Dong et al. [25] leads to a mean of 0.85% deep SSIs with cefazolin and 4.15% deep SSIs without. To detect this difference with a chi-square test with 80% power and a two-sided alpha of 0.05, 348 patients per group are required (696 in total). The loss to follow up of the WIFI-1 trial was only 1.5%. To be sure, a loss to follow up of 5% will be incorporated, coming down to a total of 732 patients.

In the absence of data for a reliable sample size calculation for the antibiotic concentration measurements we will conduct of a pilot study using 40 participants (around 20 per group). This should be sufficient to estimate the pharmacokinetic (PK) parameters; clearance and volume of distribution, the mean value, and its inter-individual variability. The availability of these population PK parameters allows Monte Carlo simulations in which T > MIC can be simulated for different MIC values and varying doses.

### Recruitment feasibility and consent

Since the WIFI-1 trial [14] was performed in the same centers and patients, we have a realistic estimation of the number of included patients each center will contribute and the amount of time it will take. In the WIFI-1 trial, it took 22 months to include 500 patients (from the start of inclusion), including a warm-up period. With largely the same hospitals participating in this trial and 21 that have already agreed to participate, the expectation is that it will take 32 months to include 732 patients. These participating centers have proved to be reliable partners in recruiting patients for previous multicenter trials [14, 26]. Moreover, taking the nature of the intervention into account, the patients’ willingness to participate is expected to be high. They are after all not exposed to risks other than in current practice and could potentially have a direct benefit from the intervention. The exclusion criteria are not different from those of the previously performed WIFI-1 trial and are therefore not expected to make a difference in number of included patients.

To ensure sufficient time to consider participation, the patient will be informed about the trial as soon as it is clear that there is an indication for IR surgery. This will be either in the emergency room or in the outpatient clinic. Documents are handed to the patient and the patient is asked to read the patient information letter. On the day of the surgery the patient will be asked to sign the informed consent form if not signed before that time. (Additional file 1).

Surgeons are asked by the coordinating investigator/project leader to check whether patients are included in the trial during the pre-operative assessment a day prior to surgery. Randomization is only performed after informed consent has been obtained.

### Statistical analysis

Descriptive methods will be used to assess quality of data, homogeneity of treatment groups and endpoints. Normality of the data will be assessed by visually inspecting the histograms and boxplots. Outcome measures will be analyzed using either a t-test or Mann–Whitney U test for continuous data according to the distributing of the data and a Chi-Square test or Fisher exact test for categorical data. If missing data is at random then missing data will be handled through multiple imputation with predictive mean matching for all variables if missing data is more than 10% and less than 50%.

#### Primary outcome

The primary analysis will be an intention-to-treat comparison of the proportion of patients with a SSI (90 days after IR) between intervention and control group, using a chi-square test. A sensitivity analysis will be performed correcting for stratification of academic/non-academic center, using logistic regression. The effect size will be primarily expressed in an absolute risk difference but a relative risk reduction will also be calculated. A two-sided p-value < 0.05 will be considered statistically significant. In all analyzes statistical uncertainties are expressed with 95% two-sided confidence intervals. Data-analysis will be performed blinded for the type of intervention.

#### Secondary outcomes

##### Cost-effectiveness and cost utility analysis

If a statistically significant difference in number of SSIs is found, cost-effectiveness of the intervention will be analyzed. The primary outcome in the CEA will be costs per patient free of SSI’s. The primary outcome in the CUA will be costs per QALY, which is a suitable outcome measure for health care policy making across interventions, patient populations, and health care settings. Both analyses will be performed from a societal perspective with a time horizon of 6 months, because we expect that differences in health outcomes and costs will be presented in the first 6 months after IR. No discounting on effects and costs will be done. To account for uncertainties a probabilistic sensitivity analysis will be performed. Incremental cost-effectiveness ratios will be calculated as the difference in costs per patient free of SSI and per QALY. Sampling variability in the CEA and CUA will be accounted for by bias corrected and accelerated non-parametric bootstrapping. Results will be reported along with their 95% confidence intervals and displayed graphically with cost-effectiveness planes and with cost-effectiveness acceptability curves. One-way and multi-way sensitivity analyzes will be done for the unit costs of health care, ratio of superficial to deep SSI.

##### Antibiotic concentrations

The T > MIC will be computed for each patient. Furthermore population PK analysis allows Monte Carlo simulations in which T > MIC can be evaluated for different doses and varying MIC values; e.g. 1, 2 and 8 mg/L.

##### Underlying infections

The presence of pathogens on implants will be displayed using descriptive statistics.

##### Independent predictors of SSI

Possible predictors will be identified by comparing baseline/surgical characteristics of patients with and without a SSI in univariate analysis (depending on type and distribution of data). Only characteristics clinically identified as possible risk factors will be included. These are: age, sex, weight, intoxications, comorbidities (such as Diabetes Mellitus or auto-immune disorders), antibiotic prophylaxis, previous SSI, duration of surgery, tourniquet use, incomplete implant removal, wound dressing and weight-bearing policy. All relevant characteristics (p < 0.2 in univariate analysis) identified in univariate analysis will be included in a multivariable logistic regression with stepwise backward selection using SSI as the dependent variable, to determine individual predictors of SSI.

### Handling and storage of data and documents

After randomization, patients will receive a numeric study identification number (anonymized). A subject identification code list will be solely accessible for the principal investigator and study coordinator. Furthermore, possibly identifying baseline characteristics are kept in an online, password protected database (Castor EDC [23]) with an audit trail. The source data will be stored after publication of results of the trial and kept by the project leader for 15 years after the inclusion of the last patient.

### Monitoring

The study will be monitored by the Clinical Research Unit of the Amsterdam UMC according to ICH-GCP guidelines throughout its duration by (a) BROK (basis course for clinical researchers on regulations and organization) or GCP-certified monitor(s) according to the Monitoring Plan (Additional file 1). The assigned monitor is not involved in the clinical trial as part of the trial site staff. The monitor’s qualifications, including the received GCP-training, are documented.

In addition, a Data Safety Monitoring Board (DSMB) is assigned, consisting of three independent professionals with complementing expertise (1 general surgeon, 1 anesthesiologist and 1 clinical epidemiologist).

The specific responsibilities of the DSMB are to:

- Monitor evidence for treatment harm (e.g. toxicity data, SAEs, deaths)
- Monitor efficacy data to guide recommendations for continuation of the study or early termination because of clear benefit, harm or futility
- Monitor planned sample size assumptions

An interim analysis will not be performed and there are no pre-specified stopping rules. A recommendation from the DSMB to terminate the study due to clear harm will be based on data showing a notably increase of (serious) adverse events in the intervention group. The justifications for a recommendation to terminate the study due to clear benefit will be based on the judgement of the DSMB and principal investigator.

The advice(s) of the DSMB will only be sent to the sponsor of the study. Should the sponsor decide not to fully implement the advice of the DSMB, the sponsor will send the advice to the reviewing METC, including a note to substantiate why (part of) the advice of the DSMB will not be followed.

### Harms

#### Adverse events

Adverse events are defined as any undesirable experience occurring to a subject during the study, whether or not considered related to the investigational product. All adverse events reported spontaneously by the subject or observed by the investigator or his staff will be recorded. The investigator will appreciate the severity of an event and consider whether the event is related to the study medication or not. The investigator will use clinical judgement to determine the relationship. Alternative causes, such as natural history of underlying diseases, medical history, concurrent conditions, concomitant therapy, other risk factors, and the temporal relationship of the event to the study medication will be considered and investigated. AEs unrelated to the study will be reported in the medical records, but not in the database.

#### Serious adverse events

A serious adverse event is any untoward medical occurrence or effect occurring within 14 days after administration of the investigational product/placebo that:

- results in death
- is life threatening (at the time of the event)
- requires hospitalization or prolongation of existing inpatients’ hospitalization
- results in persistent or significant disability or incapacity
- is a congenital anomaly or birth defect
- is any other important medical event that did not result in any of the outcomes listed above due to medical or surgical intervention but could have been based upon appropriate judgement by the investigator

An elective hospital admission will not be considered a serious adverse event. The investigator will report all SAEs to the sponsor without undue delay after obtaining knowledge of the events. The sponsor will report the SAEs through the web portal ‘ToetsingOnline’ to the accredited METC that approved the protocol, within 7 days of first knowledge for SAEs that result in death or are life threatening, followed by a period of maximum of 8 days to complete the initial preliminary report.

The following SAE’s will be listed in an overview list that will be submitted in an annual safety report to the METC and DSMB:

- allergic reactions
- surgical site infections requiring (re)admission or surgery
- re-admission or revision surgery related to the implant removal (diastasis of ankle joint, deep venous thrombosis)
- admission for diagnosis or therapy of a condition that existed before receipt of study agent(s) and has not increased in severity or frequency as judged by the clinical investigator

All other SAEs will be reported within a period of maximum 15 days after the sponsor has first knowledge of the serious adverse events.

### Dissemination and implementation of results

The results of the primary analysis will be shared with all main investigators in order to discuss results and subsequent conclusions and implications for clinical practice. After reaching consensus on trial results and conclusions, these are communicated to participants in-short and in clear language. To assure implementation of results an implementation plan has been made, focusing on the target group of trauma surgeons, orthopedic trauma surgeons, as well as microbiologists, anesthesiologists and pharmacists. Partly based on our previous experiences, we will use a combination of three implementation strategies: (1) an “informing strategy”, (2) a “motivational strategy” and (3) an “organizational strategy” would be most fitting for this intervention.

(1) To inform the target group we will largely rely on the conventional means of implementation, such as publication in an international journal and presenting on conferences. However, also a press release will be issued if the intervention proves to be effective, following up on the earlier article following the initial WIFI trial. Moreover, the participation of many centers (academic/non-academic) throughout the Netherlands will facilitate wide-spread knowledge and implementation of the results. With the multidisciplinary involvement in the development and conduction of this trial (trauma/orthopedic surgeons, anesthesiologists, medical microbiologists, pharmacists) we can raise awareness in different fields of health care. (2) To combine the motivational and organizational strategy, both the personal contact with main investigators and the electronic medical record system can be used. Nowadays, every hospital has a mandatory pre-operative checklist to confirm the correct patient, procedure but also the antibiotic prophylaxis (“time-out procedure”). This forces not only the surgeon and surgical staff, but also the anesthesiologist to think about the need for antibiotic prophylaxis. Since this procedure is documented in the electronic medical records, it can be used both to embed the results of this trial (pop-up with reminder to prescribe antibiotic prophylaxis) and for performance feedback.

### Authorship and publication

The study coordinator(s) will be first author on the primary manuscript and included in the list of authors in any subsequent manuscripts. The last authorship is reserved for the principal investigator. All other authors will be listed in alphabetical order. For purposes of abstract presentation and publication, any secondary publication will be discussed with all locally participating principal authors.

Publications will be in accordance with international recognized scientific and ethical standards concerning publications and authorship, including the Uniform Requirements for Manuscripts Submitted to Biomedical Journals, established by the International Committee of Medical Journal Editors. Copyrights concerning publications of the Clinical Study remain with the authors of the publication, regardless of any other provisions regarding intellectual property rights. Further specifications will be adapted to each individual study site.

The funding party will be mentioned on all publications of primary/secondary results of the trial.

## Discussion

Although previous trials [14, 25] investigating the effect of prophylactic antibiotics in implant removal were accurately designed based on the formerly active protocols, guidelines have changed. Two grams of cefazolin is the currently recommended dose of prophylaxis, mostly based on new insights in weight-based dosing. Moreover, a recent meta-analysis shows that antibiotic concentrations are lower when measured more distally in the extremity, indicating that 1 g might not be sufficient below the level of the knee [18]. If the hypothesis that 2 g of prophylactic cefazolin is effective in preventing SSI is supported by the results of this study, this would have implications for current guidelines. Combined with the high infection rate of IR which has already been proved in previous studies [2, 27], it would be sufficiently substantiated for guidelines to suggest protocolled us of prophylactic antibiotics in IR of foot, ankle, lower leg or patella. Moreover, if antibiotic prophylaxis proves to be effective in reducing SSIs, it is likely to be cost-effective, since it is a relatively cheap intervention. If antibiotic prophylaxis does not turn out to be effective, the target-site concentrations of measured in this trial will hopefully provide us with an explanation.

## Supplementary Information

**Additional file 1:** Description of planned monitoring of the study.

## Data Availability

The datasets that will be analyzed during the current study will become available from the corresponding author on reasonable request after publication of final results.

## Abbreviations

AE: Adverse event
ASA: American Society of Anesthesiologists
BMI: Body mass index
CDC: Centers for disease control and prevention
CEA: Cost-effectiveness analysis
CUA: Cost utility analysis
DSMB: Data safety monitoring board
EQ-5D-5L: EuroQuality of Life-5D
iMCQ: IMTA Medical Consumption Questionnaire
iPCQ: IMTA Productivity Cost Questionnaire
IR: Implant removal
Iv: Intravenous
METC: Medical Ethics Review Committee
MIC: Minimal inhibitory concentration
Nacl: Sodium chloride
NTR: Nederlands Trial Register
PK: Pharmacokinetic
QALY: Quality adjusted life years
RCT: Randomized controlled trial
SAE: Serious adverse event
SSI: Surgical site infection
UMC: University Medical Centre

## References

[1] Vos DI, Verhofstad MHJ, Hanson B, van der Graaf Y, van der Werken C (2012). Clinical outcome of implant removal after fracture healing. Design of a prospective multicentre clinical cohort study. BMC Musculoskelet Disord..

[2] Backes M, Schep NW, Luitse JS, Goslings J, Schepers T (2015). high rates of postoperative wound infection following elective implant removal. Open Orthop J.

[3] Brown BD, Steinert JN, Stelzer JW, Yoon RS, Langford JR, Koval KJ (2017). Increased risk for complications following removal of hardware in patients with liver disease, pilon or pelvic fractures: a regression analysis. Injury.

[4] Agodi A, Auxilia F, Barchitta M, Cristina ML, D’Alessandro D, Mura I (2017). Risk of surgical site infections following hip and knee arthroplasty: results of the ISChIA-GISIO study. Ann DI Ig Med Prev E DI Comunita.

[5] De Jong L, Klem TMAL, Kuijper TM, Roukema GR (2017). Factors affecting the rate of surgical site infection in patients after hemiarthroplasty of the hip following a fracture of the neck of the femur. Bone Jt J.

[6] Feilmeier M, Dayton P, Sedberry S, Reimer RA (2014). Incidence of surgical site infection in the foot and ankle with early exposure and showering of surgical sites: a prospective observation. J Foot Ankle Surg..

[7] Wiewiorski M, Barg A, Hoerterer H, Voellmy T, Henninger HB, Valderrabano V (2015). Risk factors for wound complications in patients after elective orthopedic foot and ankle surgery. Foot Ankle Int.

[8] Backes M, Schepers T, Beerekamp MSH, Luitse JSK, Goslings JC, Schep NWL (2014). Wound infections following open reduction and internal fixation of calcaneal fractures with an extended lateral approach. Int Orthop.

[9] Backes M, Schep NWL, Luitse JSK, Carel Goslings J, Schepers T (2015). The effect of postoperative wound infections on functional outcome following intra-articular calcaneal fractures. Arch Orthop Trauma Surg..

[10] Berrios-Torres S, Umscheid C, Bratzler D (2017). Centers for disease control and prevention guideline for the prevention of surgical site infection, 2017. JAMA Surg.

[11] Vos DI, Verhofstad MHJ (2013). Indications for implant removal after fracture healing: a review of the literature. Eur J Trauma Emerg Surg.

[12] Pot JH, van Wensen RJA, Olsman JG (2011). Hardware related pain and hardware removal after open reduction and internal fixation of ankle fracturesle. Foot Ankle Online J.

[13] Foundation on Antibiotic Policy Working Group (SWAB), Clinical Practice Guideline on *Peri-operative praphylaxis*. Bauer MP, van de Garde EMW, van Kasteren MEE, Prins J, Vos M. Last updated july 2019; last viewed december 2020, Available from: https://swab.nl/nl/peri-operatieve-profylaxe.

[14] Backes M, Dingemans SA, Dijkgraaf MGW, Van den Berg HR, Van Dijkman B, Hoogendoorn JM (2017). Effect of antibiotic prophylaxis on surgical site infections following removal of orthopedic implants used for treatment of foot, ankle, and lower leg fractures a randomized clinical trial. JAMA J Am Med Assoc..

[15] Bratzler DW, Dellinger EP, Olsen KM, Perl TM, Auwaerter PG, Bolon MK (2013). Clinical practice guidelines for antimicrobial prophylaxis in surgery. Am J Heal Pharm.

[16] Brill MJE, Houwink API, Schmidt S, Van Dongen EPA, Hazebroek EJ, Van Ramshorst B (2014). Reduced subcutaneous tissue distribution of cefazolin in morbidly obese versus non-obese patients determined using clinical microdialysis. J Antimicrob Chemother..

[17] Moine P, Fish DN (2013). Pharmacodynamic modelling of intravenous antibiotic prophylaxis in elective colorectal surgery. Int J Antimicrob Agents.

[18] Sanders FRK, Goslings JC, Mathôt RAA, Schepers T (2019). Target site antibiotic concentrations in orthopedic/trauma extremity surgery: is prophylactic cefazolin adequately dosed? A systematic review and meta-analysis. Acta Orthop..

[19] Abdel Jalil MH, Abu Hammour K, Alsous M, Awad W, Hadadden R, Bakri F (2017). Surgical site infections following caesarean operations at a Jordanian teaching hospital: frequency and implicated factors. Sci Rep..

[20] Sharareh B, Sutherland C, Pourmand D, Molina N, Nicolau DP, Schwarzkopf R (2016). Effect of body weight on cefazolin and vancomycin trabecular bone concentrations in patients undergoing total joint arthroplasty. Surg Infect (Larchmt).

[21] Yamada K, Matsumoto K, Tokimura F, Okazaki H, Tanaka S (2011). Are bone and serum cefazolin concentrations adequate for antimicrobial prophylaxis?. Clin Orthop Relat Res.

[22] Deacon JS, Wertheimer SJ, Washington JA (1996). Antibiotic prophylaxis and tourniquet application in podiatric surgery. J Foot Ankle Surg.

[23] Castor EDC. Castor electronic data capture. 2019. https://castoredc.com.

[24] Boxma H, Broekhuizen T, Patka P, Oosting H (1996). Randomised controlled trial of single-dose antibiotic prophylaxis in surgical treatment of closed fractures: The Dutch Trauma Trial. Lancet.

[25] Dong Y, Li S, Xu L, Zhang T (2018). Effect of cefazolin prophylaxis on postoperative infections for implants removal surgery of ankle. Int J Pharmacol.

[26] Dingemans SA, Birnie MFN, Sanders FRK, Van Den Bekerom MPJ, Backes M, Van Beeck E (2018). Routine versus on demand removal of the syndesmotic screw; a protocol for an international randomised controlled trial (RODEO-trial). BMC Musculoskelet Disord..

[27] Sanders FRK, Birnie MFN, Penning D, Goslings JC, Schepers T (2020). Surgical site infections following routine syndesmotic screw removal; a systematic review. J Orthop Trauma..

