# Supplementary material for: Wound infection following implant removal of foot, ankle, lower leg or patella; a protocol for a multicenter randomized controlled trial investigating the (cost-)effectiveness of 2 g of prophylactic cefazolin compared to placebo (WIFI-2 trial)
Source: BMC Surg. 2021 Feb 1;21:69. doi: 10.1186/s12893-020-01024-y (PMC7849087; doi:10.1186/s12893-020-01024-y)
Supplement: Supplementary file 1 — Additional file 1: Description of planned monitoring of the study. [file 12893_2020_1024_MOESM1_ESM.pdf]

MONITORING PLAN - Investigator Initiated Studies

This form is developed by the AMC-CRU and is the confidential information of the AMC-CRU. It is intended for use by CRU CRAs only. Nothing from this document may be copied, shared, or distributed without written authorization of the CRU.

**Study** *(title and short name)*:	Wound Infections following implant removal blow the level of the knee: the influence of 2g of prophylactic cefazolin, WIFI2
**Unique number (ABR):**	NL71051.018.19
**Sponsor/ Head of Department:**	Amsterdam UMC, location AMC/ Prof. Dr. H.J. Bonjer
**Coordinating Investigator/ Principal Investigator AMC:**	Dr. T. Schepers
**Department:**	Surgery
**Type of study:**	Medicinal
**Risk Classification:**	Negligible
**Monitoring Plan version and date:**	Version 1.0, dated 04DEC2019
**Appointed CRA:**	Y. Groot

Table of contents

1 Purpose of Monitoring Plan 3
2 Clinical Research Unit Service 3
3 Estimated Enrolment and Participating sites 3
4 Monitoring Visit Schedule/ Monitoring Frequency 4
4.1 Initiation Visit 4
4.2 Monitoring Visits 4
5 Interview on Location 4
5.1 Initiation Visit/ First Monitoring Visit 4
5.2 Ongoing Monitoring Visits 5
5.3 Site Close Out Visit/ Remote Site Close Out 5
6 Trial Master File/ Investigator Site File 5
7 Informed Consent Process 6
7.1 Patient Information Form (PIF) 6
7.2 Informed Consent Form (ICF) on presence 6
7.3 Informed Consent Process 6
8 In- and Exclusion Criteria 6
9 Source Data Review and Source Data Verification 7
9.1 Source Documents 7
9.2 Source Data Review (SDR) 7
9.3 Study Procedures 7
9.4 Source Data Verification (SDV) 7
9.5 Data validity/ integrity 7
9.6 Privacy law 8
10 Safety Reporting 8
11 Investigational Medicinal Product 8
12 Equipment and Facilities 8
13 Laboratory 9
14 Pharmacy 9
15 Closing and Reporting 9
15.1 Resolution of reported monitoring issues/ findings 9
16 Approval Form 10

Clinical Research Unit

1 Purpose of Monitoring Plan

Primary objective of this Monitoring Plan is to support the conduct of investigator initiated studies. This Monitoring plan facilitates compliance with the Human Research Act (WMO), Good Clinical Practice (ICH-GCP) guidelines (5.18.1) and/ or ISO14155, which require monitors to verify that:

(a) the rights and well-being of human subjects are protected.
(b) recorded study data is accurate, complete, and verifiable with the source documents.
(c) the conduct of the study is compliant with the currently approved protocol and with applicable laws regulatory requirements, e.g. WMO, ICH-GCP, ISO14155.

This Monitoring Plan should be used by the Clinical Research Associates (CRAs) while monitoring investigational sites that participate in this study. This document describes key monitoring activities and specifies the data to be verified over the course of the clinical study. These activities are in principle based on the additional risk of this study compared to standard treatment and are described in the NFU-guideline '*Kwaliteitsborging van Mensgebonden Onderzoek*' *version 3, dated March 2019.*

The study-specific Monitoring Plan will be updated and revised as needed. The reason for change will be described in an addendum. The most recent version of the Monitoring Plan will take precedence over any previous version(s).

Version control will be conducted according the SOP: Management of Quality Documents (GEN 001). All final and signed versions will be filed in the Trial Master File and the CRU Study File.

2 Clinical Research Unit Service

To support clinical researchers in complying with quality requirements, the Clinical Research Unit (CRU) was established in 2006. The primary intent of the Service is to support the conduct of investigator-initiated trials/studies and focus on compliance with WMO/GCP guidelines and local law and regulations, regardless of funding source.

Monitoring is a fee for service offered by the CRU. For the monitoring by the CRU, all parties agree on the costs, which are based on the assumptions described in paragraph 4.

The CRU CRAs involved have received at minimum an internal training specifically designed for the tasks that will be performed. All CRU CRAs have received training in the principles of WMO/GCP and have been certified therefore. The Coordinating PI is responsible for providing specific therapeutic area and/ or protocol training as well as training in the electronic Case Record Form (eCRF), if another system than Open Clinica or Castor EDC will be used.

3 Estimated Enrolment and Participating Sites*

The CRA verifies, documents and reports the enrolment rate and the percentage of drop-outs (withdrawn and lost to follow-up).

Estimated enrolment, according to applicable approved protocol.
In total 732 subjects will be included; 366 subjects per treatment arm.
Inclusion duration: 32 months
Estimated start of study: NOV2019
Estimated First Subject enrolled: NOV2019
Study participation duration for subject: 6 months
Estimated Last Subject enrolled: JUN2022
Estimated Last Subject Last Visit: DEC2022

Final database lock: MAR2023

Clinical Research Unit

*

4 Monitoring Visit Schedule/ Monitoring Frequency

The CRA will contact the site for planning of the monitoring visits conform paragraph 4.2.

4.1 Initiation Visit

An Initiation Visit is required for each participating site, including the AMC. It is expected that, at a minimum, the local PI and a study coordinator will attend this visit. Additionally, involved co-investigators and other study personnel are expected to attend this visit as well.

The responsible Coordinating PI of the AMC or delegate will perform the Initiation Visit(s) either in person or will provide all participating sites with the necessary documents and have a web-based training and/ or teleconference. A Site Initiation Training/ Attendance Log will be completed during the visit including all the personnel present at the initiation visit. The original will be kept in the ISF and a copy will be taken for the TMF. If a member of the investigator team is not present at the initiation visit, he/she will be trained by one member of the team present at the visit. This training will be documented in the Site Training Log.

4.2 Monitoring Visits

Visit no.	Selected Sites	Planning*
Initiation Visit	*All*	*Before enrolment of the first subject, but after Ethics Committee approval, declaration of "no objection" from the CCMO and approval of the Board of Directors has been obtained.*
First Monitoring Visit	All	After 3-5 enrolled subjects, irrespective of (e)CRF completion.
Second Monitoring Visit	AMC	After 50 enrolled subjects in total or one year after the first monitoring visit.
Third Monitoring Visit	All	After 50% of subjects are enrolled at site.
Extra Monitoring Visit, if needed	All	Additional Site Monitoring Visits may be required at a particular site depending on site enrolment rate, identified quality issues and/ or site performance (2 in total).
Close out visit	AMC	After last subject last visit
Remote Close Out	Participating sites	*Remote Close Out* After database lock

*The frequency may be changed based on the total enrolment period, the inclusion rate, quality issues and/ or site performance, but only after consultation with the Coordinating PI.

5 Interview on Location

The follow items will be discussed/ verified by the CRA during the different visits.

5.1 First Monitoring Visit

- Who is/ are the contact person(s) at site
- Is the entire investigators' study staff adequately informed about the study e.g. randomisation procedure, sample collection, procedures in case of protocol deviations/ serious breaches, SAE notification procedures etc.

Clinical Research Unit

- Is the entire investigators' study staff WMO/GCP trained and authorized (site signature and delegation log)
- Has the study staff sufficient time to perform the study?
- How and by whom is the subject informed about the study?
- By whom is consent obtained and is it properly documented?
- Who will examine the subject every visit?
- Who performs the screening, baseline and other visits/ how is this arranged?
- Which source documents are available?
- Where is the source data stored?
- Who will maintain the subject identification code list/ screening log/ enrolment log?
- Who is completing the (e)CRF?
- What are the procedures with regard to the dispensing of investigational medicinal product, investigational medicinal product accountability, the return of investigational medicinal product, unused investigational medicinal product and unblinding procedure?
- Where is/ are the investigational medicinal product(s) stored and who has access?
- How is temperature of investigational medicinal product(s) monitored?
- When/ how/ where and by who are questionnaires filled in?
- Have any Serious Adverse Events (SAEs)/ Suspected Unexpected Serious Adverse Reactions (SUSARs) occurred?
- Are there any known protocol deviations and/ or serious breaches of ICH-GCP and/ or protocol?
- Is the Trial Master File/ Investigator Site File up to date (AMC SOP CTR 006/ ICH-GCP guideline 8.1 – 8.3)?
- What is the expected recruitment rate?
- Competitive studies running?

5.2 Ongoing Monitoring Visits

- Is the entire investigators' study staff adequately informed about the study?
- Is the entire investigators' study staff WMO/GCP trained and authorized (site signature and delegation log)
- Are there any changes in the investigators' study staff (trained and authorized)?
- Are there any changes in facilities or equipment?
- Is the investigational medicinal product accountability properly documented?
- Have any SAEs/ SUSARs occurred?
- Are there any known protocol deviations and/or serious breaches of ICH-GCP and/or protocol?
- Is the Trial Master File/ Investigator Site File up to date (AMC SOP CTR 006/ ICH-GCP guideline 8.1 – 8.3)?
- Are there any new amendments in place?

5.3 Remote Site Close Out

No onsite Site Close Out Visit will be performed, but a Remote Site Close Out checklist will be send to the site for completion. The checklist will be returned to the CRU, signed and dated by the local PI to confirm the study closure onsite.

6 Trial Master File/ Investigator Site File

The CRA assesses the Trial Master File/ Investigator Site File according to AMC SOP CTR 006 and ICH-GCP guideline 8, and evaluates how essential documents are administered. The CRA specifically verifies the presence of:

- The Ethics Committee approval(s) (including membership list)
- CCMO (Central Committee on Research Involving Human Subjects) notification(s) of no objection
- Annual progress reports and annual safety reports/ drug safety update reports
- Completed SAE Forms and documentation on the date(s) of reporting

- A completed and up to date subject screening and enrolment log
- A completed and up to date subject identification log
- Qualification of the investigators' study staff (CV, WMO/GCP training, protocol training)
- A completed and up to date site signature and delegation log
- A valid WMO insurance certificate

7 Informed Consent Process

7.1 Patient Information Form (PIF) and Informed Consent Form (ICF)

The CRA verifies if:

- the approved version of the PIF/ICF is used
- the PIF contains the proper investigational site-specific details
- consent for access to medical records for the CRA/ Auditors/ Authorities (IGJ, EMA, FDA) is given

7.2 Informed Consent Form (ICF) on presence

The CRA verifies for an at random selection of at least 25% of the randomised subjects if a signed ICF is present.

7.3 Informed Consent (IC) Process

The CRA verifies the ICFs for an at random selection of at least 10% of the randomised subjects on the following IC process items:

- is the approved version of the ICF used?
- is the ICF personally signed and dated by the subject or by the subjects' legally acceptable representative?
- is the ICF signed and dated prior to any study related procedure?
- are exceptions documented and approved by the Ethics Committee?
- is the ICF personally signed and dated by a witness, if applicable?
- is the ICF personally signed and dated by the member of the study staff who informed the subject about the study and performed the consent process?
- did the investigator or delegate sign and date the ICF at the same time as the subject or the subjects' legally acceptable representative?
- is there documentation that a copy of the PIF and the signed ICF has been given to the subject or the subjects' legally acceptable representative?
- is the subjects' participation documented in the medical records?
- is the signed original ICF filed in the Investigator Subject Site File or a separate study binder?
- has the revised PIF/ ICF been signed by all active subjects in a timely fashion, if applicable (re-consent)?

8 In- and Exclusion Criteria

The CRA verifies **all** in- and exclusion criteria for an at random selection of at least 10% of the randomised subjects at site. These in- and exclusion criteria to verify are documented in the applicable approved protocol.

If during the course of the study a subject is included who does not fulfil the in- and exclusion criteria and there is no documentation present that this deviation was accepted by the Coordinating PI, the planned 10% of study subjects to verify will be increased for that specific site in consultation with the Coordinating PI and the Monitoring Plan will be updated accordingly.

9 Source Data Review and Source Data Verification

9.1 Source Documents

- Paper medical records
- EPD system/ hospital local electronic patient record system
- Study related paper work sheets
- Subject questionnaires
- Investigational (medicinal) product accountability forms
- Other: collection of blood & soft tissue (AMC only)
- Cost-effectiveness of intervention: measured with health care resource utilization and costs (iMCQ, iPCQ; at baseline, 6 weeks, 3 months and 6 months after surgery.
- Quality adjusted life years (QUALY's), measured by the 5-level EuroQuality of Life -5D (EQ-5D-5L), at baseline, 2 weeks, 6 weeks, 3 months and 6 months after surgery.

9.2 Source Data Review (SDR)

For all subjects selected for Source Data Verification (9.4), Source Data Review will be performed, i.e. protocol compliance will be checked. For all protocol deviations, the local PI is obliged to maintain an overview. For multicentre studies the Coordinating PI must approve or acknowledge all protocol deviations.

9.3 Study Procedures

The CRA verifies if:

- study related procedures are described e.g. '*noodprocedure*' in case of electronic randomisation systems
- the current approved version of the protocol (including amendments) has been adhered to by the study staff
- the investigator and other site staff are performing study related activities in accordance with the site signature and delegation log
- protocol deviation logs are maintained, complete and filed

9.4 Source Data Verification (SDV)

The CRA verifies whether the study data entered in the (e)CRF is accurately reported and consistent with the source documents for an at random selection of at least 10% of the randomised subjects. Please note that this population selected might differ from the population selected for the check on in- and exclusion criteria.

The data (a predefined list of variables, including the primary endpoint) to verify as determined in consultation with the Coordinating PI:

- general demographic data (such as age, gender) at baseline
- in- and exclusion criteria
- source documents are accurate, complete, kept up to date and maintained
- medical history and current medical conditions
- primary endpoint: *Surgical Site Infection (SSI) within 90 days , as defined by criteria used in the latest CDC guideline of the prevention of SSI.*
- documentation of AEs/ SAEs/ SUSARs

In case of transcription errors in eCRF, the planned amount subjects to verify might be increased for that specific site, in consultation with the Coordinating PI, and the Monitoring Plan will be updated accordingly.

9.5 Data validity/ integrity

The CRA checks if the type of database used to collect and analyse study data is compliant with ICH-GCP/ ISO14155 and the AMC RDM SOPs, as applicable.

9.6 Privacy law

The CRA verifies if:

- the study is registered to the AMC *"Functionaris Gegevensbescherming (FG)"*
- all personal identifiable records (e.g. name, date of birth, address and medical records number) are kept in a separate binder, Investigator Subject Site File

10 Safety Reporting

SAEs/ SUSARs will be collected and recorded throughout the study period, defined as informed consent signature to week 2.

The CRA verifies:

- presence of unreported SAEs/ SUSARs for the subjects selected for SDV/ SDR
- whether all SUSARs and at least 10% of the reported SAEs are recorded accurately and within the time frames required by the protocol, ICH-GCP and the applicable law/regulations
- whether all SUSARs and at least 10% of the reported SAEs have been reported to the Sponsor/ Coordinating PI within the time frames required by the protocol
- whether all SUSARs and at least 10% of the SAEs reported to the Sponsor/ Coordinating PI have been reported to the Ethics Committee that approved the study and if applicable to the Competent Authority, as per protocol and within the time frames required by ICH-GCP and the applicable law/regulations.

If more than 3 SAEs are reported to the Ethics Committee that approved the study, but not within the required time frame, attention will be paid to the reporting process.

In case one unreported SAE is discovered, the CRA will additionally review, at that particular Monitoring Visit and if the source documents are available, the source documents of 2 randomised subjects not selected for SDV/ SDR, for any unreported SAEs. If one or more unreported SAE is discovered, an additional Monitoring Visit will be planned in consultation with the Coordinating PI, to check all source documents of the subjects randomised so far for unreported SAEs.
In case unreported SAEs are discovered, the investigator is requested to report them as mandated by the protocol and re-training of site-staff by local PI is strongly recommended.

11 Investigational Medicinal Product

The CRA verifies if:

- the study staff is adequately trained in the randomisation procedure of subjects and obtaining the investigational medicinal product assignment
- the prescription by the investigator is in place
- the investigational products are administered to eligible subjects only and subjects received the protocol specified dose(s)
- the expiry date and lot/ batch/ serial number of the investigational medicinal products are documented
- the investigational medicinal product accountability is complete, accurate and consistent with other documentation
- there is an unblinding procedure in place
- temperature logs of the stored investigational (medicinal) products are in place in the case of storage at a location other than the pharmacy.

12 Equipment and Facilities

NA

13 Laboratory

The CRA verifies (AMC only):

- the correct labelling of the samples
- the protocol specific requirements for sample storage are adhered to

14 Pharmacy

The CRA verifies:

- the received prescription forms are available
- the investigational medicinal product accountability forms are available, complete and accurate.

15 Closing and Reporting

After every Monitoring Visit at a site, the CRA will write a Site Monitoring Visit Report. This report will summarize the tasks performed by the CRA. It will contain a general description of the quality of the site and an overview of all issues/ findings/ discrepancies with an advice for corrective actions. The original signed and dated Site Monitoring Visit Report will be sent to the Head of the Department and the Coordinating PI. This report will have to be read and filed in the Trial Master File.

For multicentre studies, a copy of the Site Monitoring Visit Report will also be sent to the site and/ or location visited. Also this report will have to be read, filed in the Investigator Site File (at site/ location visited) and the Trial Master File (sponsor).

In case of an audit or inspection Monitoring Reports should be made available.

15.1 Resolution of reported monitoring issues/ findings

Adequate and timely follow-up of all issues/ findings/ discrepancies must be performed by the study staff. Repeated protocol deviations, serious breaches and/ or high priority issues must be resolved or action must be initiated within 4 weeks upon receipt of the Site Monitoring Visit Report. Low priority issues should be resolved or action should be initiated preferably within 10 weeks, but at least prior to the next Monitoring Visit.

Issues graded as high priority issues must be resolved or action must be initiated within 4 weeks. After all issues with high grading have been resolved or action has been initiated , the completed and signed 'Monitoring Issues and Action Tracker' (appendix to the Site Monitoring Visit Report) must be returned to the CRA (as PDF). If the issues are not resolved or no action has been initiated within 4 weeks a reminder will be sent to the responsible Principal Investigator. If no action has been taken in response to the reminder within 2 weeks, the 'Monitoring Issues and Action Tracker' will be forwarded to the AMC QA Manager and Manager QC&S.

Follow-up of all issues/ findings/ discrepancies will be verified by the CRA during the next Monitoring Visit, or earlier as applicable.

Remote Site Close Out

In case of unconfirmed outstanding issues/ findings/ discrepancies at the time of a Remote Site Close Out , they will be communicated and the investigators' confirmation for the resolution of the outstanding actions is requested. A listing of the outstanding issues/ findings/ discrepancies is added to the concerned Remote Site Close Out Checklist. The completed and signed documents must be returned to the CRA (as PDF) and filed in the Investigator Site File or Trial Master File, as applicable.

16 Approval Form

The Principal Investigator will permit monitoring and make time available to meet with the CRA on a regular basis to discuss the progress of the study. In case of non-response from the participating local PI the Coordinating PI/designee will be contacted.

Additionally, the Principal Investigator will only delegate study related tasks to qualified persons. Furthermore, the Principal Investigator confirms that read only and verification access to the eCRF is permitted for the CRA.

By signing this Monitoring Plan the Coordinating Principal Investigator agrees upon following the applicable WMO/ ICH-GCP/ ISO14155 guidelines and he/ she will make sure that appropriate measures will be taken in case of high priority issues, recurrent protocol deviations and/ or serious breaches.

I have read this Monitoring Plan. I approve this document and I agree that it contains all necessary details for monitoring as described.

Author: *Y. Groot, Sr. CRA & Quality Consultant*

Signature Date

04DEC 2019

Review: *S. Camic, Manager Quality Control & Support*

Signature Date

05dec2019

Coordinating Principal Investigator: *T. Schepers, MD. PhD*

Signature Date

9/12/2019

Head of Department/ Sponsor: *Prof. Dr. H.J. Bonjer*

Signature Date

13/12/'19

17 Appendix I: Participating Sites

Participating site	Amount of planned subjects	Name contact person/ role	Contact details: phone/ e-mail
Amsterdam UMC, location AMC	108	F.R.K. Sanders / PhD student<br>D. Penning/ PhD student	020-5660260 / f.r.sanders@amc.nl<br>Wifi2@amc.uva.nl
Alrijne Ziekenhuis	52	Ritchie, E.D., local PI	<edritchie@alrijne.nl>
Amphia Ziekenhuis	34	Vos, D.I. , local PI	DVos@amphia.nl
Amsterdam UMC, location VUMC	13	Bloemers, F.W. (Frank) , local PI	<fw.bloemers@amsterdamumc.nl>
Amstelland Ziekenhuis	12	Garssen, Frank, local PI	<frgar@zha.nl>
BovenIJ Ziekenhuis	26	Bas Twigt, local PI	<B.Twigt@BovenIJ.nl>
Catharina Ziekenhuis	53	Alexander van der Veen, local PI	<alexander.vd.veen@catharinaziekenhuis.nl>
Deventer Ziekenhuis	32	Roerdink, W.H. , local PI	<Roerdink@dz.nl>
Elkerliek Ziekenhuis	29	Jan Bernard Sintenie, local PI	<sintenie@hotmail.com>
Flevo Ziekenhuis	34	Dijkman, Bart van, local PI	<BvDijkman@Flevoziekenhuis.nl>
Franciscus Gasthuis & Vlietland	6	Beek, Flip van, local PI	<F.vanBeek@franciscus.nl>
Haaglanden Medisch Centrum Location Westeinde and Bronovo	44	Jochem Hoogendoorn, local PI	<j.hoogendoorn@haaglandenmc.nl>
Jeroen Bosch Ziekenhuis	14	Zwaard, Babette van der, local PI	<B.v.d.Zwaard@jbz.nl>
Maasstad Ziekenhuis	31	Schep, N.W.L. (Niels) , local PI	<SchepN@maasstadziekenhuis.nl>
MUMC	12	Poeze M. (Martijn) , local PI	<m.poeze@mumc.nl>
Noordwest Ziekenhuis groep Location Alkmaar	33	Joosse, Pieter, local PI	<P.Joosse@nwz.nl>
OLVG Location Oost and West	32	Veen, Ruben van, local PI	<r.n.vanveen@olvg.nl>
Reinier de Graaf Groep	6	Vries, M.R. de, local PI	<M.deVries@rdgg.nl>

Spaarne Gasthuis (location Haarlem and Hoofddorp)	109	Sosef, Nico, local PI	<NSosef@spaarnegasthuis.nl>
UMC Groningen	12	El Moumni, M, local PI	<m.el.moumni@umcg.nl>
Dijklander Ziekenhuis, location Hoorn	40	Winkelhagen, Jasper, local PI	<J.Winkelhagen@westfriesgasthuis.nl>

Addition of new sites, change of PI/ contact details is not an reason for revision of the Monitoring Plan. Addendum with new contact details should be attached to the applicable Monitoring Plan.
